# Evaluation of the Dental Caries Status of New Pediatric Patients in Tertiary Dental Institutions in 2013, 2018, and 2023

**DOI:** 10.3390/children12080960

**Published:** 2025-07-22

**Authors:** Eimi Tabata, Ami Kaneki, Masashi Ogawa, Taku Nishimura, Yuya Ito, Shunya Ikeda, Yasuko Tsuge, Shuma Hamaguchi, Tatsuya Akitomo, Ryota Nomura

**Affiliations:** Department of Pediatric Dentistry, Graduate School of Biomedical and Health Sciences, Hiroshima University, Hiroshima 734-8553, Japan; amytaba1@hiroshima-u.ac.jp (E.T.); kaneki@hiroshima-u.ac.jp (A.K.); caries0@hiroshima-u.ac.jp (M.O.); nishi04@hiroshima-u.ac.jp (T.N.); yuuya@hiroshima-u.ac.jp (Y.I.); shunyaikeda@hiroshima-u.ac.jp (S.I.); tsuge08@hiroshima-u.ac.jp (Y.T.); syuumai@hiroshima-u.ac.jp (S.H.); rnomura@hiroshima-u.ac.jp (R.N.)

**Keywords:** dental caries, pediatric dentistry, tertiary dental institutions

## Abstract

Background: Although the prevalence of dental caries in children has been decreasing in recent years, many patients still visit tertiary dental institutions with dental caries as their chief complaint. In addition, the COVID-19 pandemic that occurred around the world in 2020 may have affected the lifestyle and oral condition of children. Methods: We investigated the oral condition of new patients whose chief complaint was dental caries, and compared the results for 3 fiscal years: 2013, 2018, and 2023. The caries status was evaluated using the dmft/DMFT index. Results: The number of subjects was 129 in 2013, 163 in 2018, and 127 in 2023. The number of preschoolers in 2023 was lower than in the other years, whereas the number of elementary school students was higher, showing a statistically significant difference (*p* < 0.05). For the 3 years investigated, there was no change in the incidence of dental caries in primary teeth in elementary school children; however, the incidence in permanent teeth increased over time. Additionally, in the age group, the df and DMF scores were higher in 2023 than in the other years. Conclusions: At this tertiary dental institution, the number of elementary school patients, especially females, increased over the 10-year period, and the amount of dental caries in their permanent teeth also increased. It is important for dental professionals to understand this trend and focus on providing oral hygiene education to this age group.

## 1. Introduction

Dental caries is the most common infectious disease, affecting approximately 60–90% of the global population, especially young children [1]. Maintaining oral function is important in terms of biting, chewing, smiling, speaking, and psychosocial wellbeing. Therefore, childhood caries is a major problem not only for oral health but also for general health [2,3].

Over the past few decades, the incidence of dental caries has declined worldwide, particularly in high- and middle-income countries [4]. A Japanese nationwide survey reported that the prevalence of dental caries among children is declining, with the rate reaching an all-time low across all school types in 2024 (20.74% in kindergarten, 32.89% in elementary school, 26.50% in junior high school, 34.70% in high school) [5]. In addition, Okawa et al. (1992) reported DMFT of 3.60 in Japanese schoolchildren aged 12 years old in 1985, which was significantly lower than that in 1970–1975 (5.47) [6]. These results suggest that the prevalence of dental caries has been decreasing over time in educational institutions. However, dental caries remains the most common complaint among patients visiting tertiary dental teaching institutions in Japan [7,8,9,10]. We previously investigated new preschool patients with a chief complaint of dental caries in our hospital and found that the number increased year by year [11]. However, this study did not include the dental caries status of school age or older children, and there are few reports examining the caries status of pediatric patients of various ages over time in dental institutions.

COVID-19 was first detected in China in December 2019 and was declared as a pandemic by the World Health Organization on 11 March 2020 [12]. The first case of COVID-19 infection was confirmed in Japan on 16 January 2020, and the Japanese government ordered the temporary closure of all Japanese elementary, junior high, and high schools at the end of February to avert the pandemic [13]. The COVID-19 pandemic resulted in numerous changes in school-aged children’s daily lives, including the wearing of masks, social distancing recommendations, increased time spent at home, and the cancellation of large-scale school events [14]. These social changes could also have affected the oral condition of pediatric patients.

We investigated new patients with a chief complaint of dental caries in 3 separate years, each 5 years apart (2013, 2018, and 2023). The survey period included the COVID-19 pandemic. The purpose of this study was to analyze the longitudinal changes in the caries status of pediatric patients in tertiary dental institutions by age and to clarify the oral condition of children in recent years.

## 2. Materials and Methods

### 2.1. Subjects

Almost 500 new pediatric patients come to our hospital every year. The study used a cross-sectional study design, and subjects who met the two criteria described below were selected using complete enumeration. The first inclusion criteria are patients who visited our pediatric dental department for the first time each of the 3 fiscal years: 2013, 2018, and 2023, and excluded patients who had visited our department in the past. In our department, the chief complaints of new patients are recorded based on the contents of the referral letter, and the complaints of the patient or their guardian are recorded. The second criterion is patients whose chief complaint was dental caries.

We compared the subjects in 3 fiscal years: 2013, 2018, and 2023. In addition, we classified them into three groups: preschoolers, elementary school students, and junior or high school students.

### 2.2. Evaluation of Dental Caries

This study was conducted at the pediatric dentistry of the Hiroshima University Hospital, and intraoral examinations were performed in accordance with previously described methods [15]. In brief, pediatric dental specialists from the Japanese Society of Pediatric Dentistry examined the children, using dental mirrors and probes. Radiographic examinations were optional in this study because it was difficult to take more radiographs of all teeth in all patients to assess the presence and severity of dental caries than would be necessary in clinical practice.

We used the decayed, missing, and filled teeth (dmft/DMFT) index, which is the most widely used caries index and recommended by the World Health Organization (WHO) as an operational definition [16]. The detailed status of each tooth was evaluated in accordance with a Japanese national survey, with classifications of normal, decayed, missing, or filled teeth [17]. Decayed tooth was defined as obvious caries cavities, demineralized/eroded enamel, softened pits and fissures, and lesions on smooth surfaces [17]. Missing primary teeth were not included because it is difficult to distinguish between loss due to dental caries and natural physiological exfoliation.

The caries status of each patient was determined and compared for each year (primary teeth in preschoolers, primary and permanent teeth in elementary school students, and permanent teeth in junior high school or high school students).

### 2.3. Statistical Analysis

Statistical analyses were conducted using GraphPad Prism 9 (GraphPad Software Inc., La Jolla, CA, USA). The Mann–Whitney U test was used to compare the dental caries status between the groups. Differences were considered statistically significant at *p* < 0.05.

## 3. Results

### 3.1. Subjects

A breakdown of the subjects in this study is shown in Table 1. Of the 500 new patients who attended our hospital every year, the number of patients whose chief complaint was dental caries was 129 (28.0%) in 2013, 163 (32.0%) in 2018, and 127 (23.5%) in 2023. The prevalence in 2023 was the lowest in 3 years and showed significant difference compared to that in 2018 (*p* < 0.01).

We subdivided the patients into three categories: preschoolers; elementary school students; and junior and senior high school students. The prevalence of preschoolers was the highest in all 3 years; however, there were more elementary school students in 2023 than in other fiscal years, showing a statistically significant difference (*p* < 0.05). The number of junior and senior high school students did not change (approximately 5%).

### 3.2. Preschoolers

The results for preschoolers are shown in Table 2. Although no sex differences were found in 2013, the proportion of males increased over time, reaching 52.5% in 2018 and 53.7% in 2023. The number of decayed teeth (d) was highest in 2023 (9.07 ± 4.87) and lowest in 2018 (8.00 ± 4.80); however, no secular change was observed. The number of filled teeth (f) increased over time, from 0.32 ± 1.43 in 2013, to 0.55 ± 2.04 in 2018, and 0.82 ± 2.35 in 2023. The number of decayed and filled teeth (df) was similar to that of d.

### 3.3. Elementary School Students

The results for elementary school students are shown in Table 3. The proportion of males was over 60% in both 2013 and 2018, but the proportion decreased over time, and in 2023, more than half were females.

In the primary teeth, the d score remained steady at approximately four throughout the 3 years, with no obvious change. However, the f score increased over time (0.19 ± 0.89 in 2013, 0.89 ± 1.52 in 2018, 1.08 ± 1.90 in 2023) and that in 2013 showed a statistically significant difference compared in 2018 or 2023 (*p* < 0.01). The df score also increased accordingly (4.22 ± 3.26 in 2013, 5.16 ± 4.25 in 2018, 5.23 ± 4.44 in 2023).

In the permanent teeth, there was a marked increase in the decayed teeth (D) score and the decayed, missing, and filled permanent teeth (DMF) score over time (D score: 1.08 ± 1.71 in 2013, 1.32 ± 1.83 in 2018, 1.96 ± 2.99 in 2023/DMF score: 1.14 ± 1.78 in 2013, 1.89 ± 2.06 in 2018, 2.36 ± 3.13 in 2023), and the DMF score in 2013 showed significantly difference compared to other years (*p* < 0.05). In addition, the number of filled teeth (F) in 2018 (0.58 ± 1.22) and 2023 (0.30 ± 0.77) was also significantly higher than that in 2013 (0.06 ± 0.33) (*p* < 0.05).

### 3.4. Junior and Senior High School Students

In 2023, two patients with insufficient detailed information were excluded from the dental caries data, and only four patients’ data were shown in Table 4. The sample size for junior and senior high school students was small, and the results varied widely from year to year. The F score showed an increase over time (0.00 ± 0.00 in 2013, 1.14 ± 2.19 in 2018, 1.43 ± 2.15 in 2023).

## 4. Discussion

In clinical pediatric dentistry, dental professionals encounter patients with a variety of dental problems, including malocclusion, fused teeth, supernumerary teeth, congenitally absent teeth, and hypomineralized teeth [18]. However, dental caries remains a challenge as the most common primary complaint among pediatric patients [7,8,9,10]. Many studies have reported on dental caries status in pediatric patients; however, most studies have been conducted over a single time period or have used data from nationwide surveys [19,20,21]. We investigated oral conditions over time in multiple age groups during childhood in tertiary dental institutions.

Although the number of new patients in our hospital increased year by year, the number of patients whose chief complaint was dental caries peaked in 2018 and had decreased by 2023. As mentioned above, the number of patients with dental caries was decreasing, which may account for these results. Other factors contributing to this trend include the increasing variety of complaints from pediatric patients, and the fact that this hospital has a medical department, which means that patients with a variety of systemic diseases, such as pediatric cancer or hemophilia, attend our clinic [15,22].

We added a comparison of caries patients by age to clarify the changes in the status of patients with caries. In 2023, the proportion of preschoolers with caries decreased, and the proportion of elementary school students with caries increased; both these differences were statistically significant when compared with other fiscal years (*p* < 0.05). These findings suggest that the relative proportions of children with dental caries in tertiary dental institutions have changed over the past 10 years.

### 4.1. Dental Caries in Preshoolers

No significant changes were observed in the oral condition of preschoolers over the 3 fiscal years, including sex differences. Traditionally, dental caries in preschoolers have been called various names, such as “rampant caries” and “baby bottle syndrome,” but now all clinical manifestations have been joined and classified as early childhood caries (ECC) [23]. Zaror et al. (2022) investigated the association between ECC and oral health-related quality of life (OHRQoL), and concluded that ECC has a negative impact on the OHRQoL of both preschoolers and their families [24]. The responsibility for young children’s health usually belongs to their parents, who usually make the decisions about their health [25]. These findings highlight the importance of educating the parents and providing appropriate dental intervention for ECC even if the preschoolers do not understand the importance of dental treatment or are uncooperative with dental treatment. In Japan, the number of dual-income households is increasing year by year, and parents accompanying pediatric patients often also work [26]. The parents of children with ECC may have to take time off work to stay home and care for their children or spend time and money to have the disease treated [25]. Thus, parents’ circumstances may also affect the preschoolers’ dental visits. Gomes et al. (2021) reported that a history of dental pain affects the OHRQoL of preschoolers, and that early diagnosis of dental caries and the implementation of treatment should occur before there is an impact on OHRQoL [27]. Regular dental checkups help prevent dental caries, and even if dental caries do occur, early dental intervention leads to improvement in the OHRQoL of preschoolers.

### 4.2. Dental Caries in School-Age Children

In contrast to the results for preschoolers, the dental caries experience of elementary school students changed and the condition, especially in permanent teeth, worsened over time. Ota et al. (2013) found that of 6069 elementary school students, more than 80% brushed their teeth two or more times a day [28]. The Japanese Society of Pediatric Dentistry recommends toothbrushing by the parents between the ages of 8 and 10 [29]. However, in a survey conducted by Sato (2009), 78.5% of the parents of elementary school students stated that they rarely or never brushed their children’s teeth [30]. Although the habit of brushing teeth has become established among children, immature brushing techniques and insufficient finishing brushing may affect the occurrence of dental caries in permanent teeth. In recent years, toothpaste containing fluoride has become popular in Japan, and the use of toothpaste containing 1500 ppm fluoride is recommended for pediatric patients aged 6 and over [31]. Abuhaloob et al. (2023) found that a comprehensive school oral health program based on school-health education combined with supervised toothbrushing using 1450 ppm fluoride toothpaste was efficacious [32]. Appropriate health education in schools can lead to a reduction in dental caries among people of this age group.

Although the sample size was small, multiple caries were detected in junior and senior high school students. The permanent dentition stage in childhood is the time when children learn to become independent from their parents in their oral health habits, and our previous study showed that it is more difficult to improve oral health habits in patients with permanent dentitions than in those in the mixed dentition stage [15]. Reddy et al. (2020) investigated the risk factors for dental caries in children aged 13–17 years, and found associations with the quantity, low buffering capacity, and low pH of stimulated saliva, and the consumption of sugary food [33]. It is important for dental professionals to understand these risk factors and provide intensive oral health education for high-risk groups to achieve a reduction in dental caries in permanent teeth.

### 4.3. Sex-Based Differences

Comparing the results in 2023 by gender, although the number of males was more than that of females in preschoolers, females were more in the elementary school and above categories. In particular, the proportion of females in elementary school has increased over time. Mattila et al. (2016) investigated oral health-related knowledge, attitudes, and habits among 12-year-olds, and found that girls reported a higher percentage of health promotional habits than boys [34]. However, many studies have reported a higher prevalence of dental caries in girls [35,36,37,38]. Yamada et al. (2025) concluded that both biological and anthropological factors such as sex-based dietary preferences may cause sex differences [38]. Further additional research is needed to clarify these differences between males and females.

### 4.4. Impact of the COVID-19 Pandemic

The present study was carried out over 3 fiscal years, with the COVID-19 pandemic occurring in 2020. The COVID-19 lockdown affected the daily oral hygiene routines of children, potentially increasing caries risk [39]. Mirhashemi et al. (2022) reported that the stress caused by the COVID-19 pandemic increased detrimental oral habits, such as bruxism as well as temporomandibular disorders in adults and adolescents, causing a negative impact on oral conditions across all age groups [40]. By contrast, Zarabadipour et al. (2023) reported that increased time spent together during lockdowns helped most parents monitor their children’s oral hygiene habits during the pandemic, improving the health of family members [41]. This finding suggests that the pandemic did not necessarily worsen oral health. In this study, the association between the pandemic and the deterioration in dental caries status over time was unclear. Additional research, such as cohort studies comparing pre- and post-pandemic data adjusting for confounding variables, is needed to clarify any association in the future.

### 4.5. Future Challenges

In Japan, a nationwide survey is conducted every five to six years. Compared to the previous survey in 2011 and 2016, the 2022 results have been decreasing with almost df scores for ages 1 to 6, with the highest value being 1.2 for ages 6 [42]. In the DMF score of 7 to 12 years, there was no significant change or a slight decrease over time, with the maximum value being 1.0 at age 11 [42]. On the other hand, in the present study, the df score in preschoolers was 9.89 ± 4.95, and the DMF score in elementary school students was 2.36 ± 3.13, both of which were higher than that of the nationwide survey. Because this survey was conducted at a medical institution, it is natural that the results were high; however, it suggests that the increase in the DMF score in elementary school students over time is a characteristic finding of this study. In addition, the f score also increased over time, indicating that patients with dental caries had received dental treatment before visiting our department. These results suggest that although the number of children with dental caries is decreasing, there are an increasing number of cases in which more older children could not manage their caries despite dental treatment in the past and were referred to tertiary dental institutions. Masiga et al. (2013) reported that children with high dmft manifested negative impacts on appearance, chewing, biting hard foods, and missing school on account of toothache and discomfort, while in the permanent dentition, children with high DMFT had a negative impact on biting hard foods, suggesting that dental caries prevention is important for children’s quality of life [43].

Regular dental checkups prevent the occurrence of dental caries [44]. However, oral management of the patient cannot be provided without dental visits, and two approaches are key for preventing dental caries. One is the approach of educational institutions. In Japan, there is a position called a school dentist, who is responsible for conducting annual dental checkups at educational institutions, which can lead to the early detection and treatment of oral diseases [45]. School dentists also play a role in health education, giving lectures to children, such as the function of the oral cavity, the important role of mastication, and the interrelation between general and oral health, together with a school dentist, a schoolteacher, or a nurse teacher in the school curriculum [45]. In children, the eruption of permanent teeth varies depending on age, and the points to pay attention to vary. Therefore, providing health education appropriate for each age group is essential. Additionally, some municipalities have reported decreased DMF scores after introducing fluoride mouthrinses in schools [46]. Dental professionals must work with educational institutions to implement effective oral health programs for all children.

The other is collaboration among dental institutions. This study ultimately increased the number of patients with filled teeth at tertiary dental institutions. The International Caries Detection and Assessment System (ICDAS) can also be used to monitor the evolution of a lesion over time, which helps in the clinical evaluation of the effectiveness of any treatment [16]. Even for pediatric patients who have completed dental treatment, it is essential to continuously evaluate the condition of their caries using ICDAS at regular visits, and to consider cooperation with tertiary dental institutions if rapid progression is observed. In addition, future research is needed at various medical institutions, such as private pediatric dental clinics and other tertiary dental institutions, to clarify trends among pediatric patients in recent years.

In our department, we provide the patient whose chief complaint is dental caries with oral hygiene education, including dietary advice at the initial visit. In addition, even after dental caries treatment is completed, patients continue to undergo regular checkups to prevent the recurrence of dental caries. We conducted a post-intervention study of the periodontal status and oral health habits of hemophilia patients who visited our clinic for five consecutive years [15]. In the study, continuous dental support improved the periodontal status in hemophilia patients in the mixed dentition stage. In this way, the post-intervention of patients is important in identifying the effectiveness of the oral health program and identifying new strategies. Most participants in this study are still trying to prevent new dental caries through regular dental visits and oral hygiene education even now. In the future, we would like to investigate the effectiveness of the oral health program for them by conducting a post-intervention survey of these participants.

### 4.6. Limitation

This study had some limitations. First, we selected subjects on the basis of a single inclusion criterion: new patients who visited our clinic with a chief complaint of dental caries. Therefore, other factors, such as systemic diseases or intellectual disability, were not accounted for in this study. Second, the oral hygiene habits of the patients were not evaluated. Additional future research should focus on oral hygiene habits across different age groups. Third, we did not evaluate reproducibility, examiner calibration, and reliability. However, in this study, pediatric dental specialists certified by the Japanese Society of Pediatric Dentistry performed oral examinations. There are approximately 100,000 dentists in Japan, but these pediatric dental specialists account for only 1.1%, comprising around 1100 individuals. In addition to conducting examinations at the same dental clinic as in our previous study on children [15], we established stricter criteria in this study, requiring pediatric dental specialists to perform intraoral examinations.

## 5. Conclusions

Although nationwide surveys have reported a decrease in dental caries, the number of elementary school students presenting at our tertiary dental institution increased significantly, especially in females. This finding suggests that there are still patients with severe caries, and reducing the numbers of these patients is an ongoing challenge for dental professionals. It is important to develop and implement oral hygiene education for each age group.

## Figures and Tables

**Table 1:** New outpatients with a chief complaint of dental caries.

Fiscal Year	2013	2018	2023
Prevalence of patients with a chief complaint of dental caries	129/460 (28.0%)	163/509 (32.0%)	127/540 ^††^ (23.5%)
Preschoolers	87 (67.4%)	118 (72.4%)	67 *^,†††^ (52.8%)
Elementary school students	36 (27.9%)	38 (23.3%)	53 *^,†††^ (41.7%)
Junior and senior high school students	6 (4.7%)	7 (4.3%)	7 (5.5%)
Total	129 (100.0%)	163 (100.0%)	127 (100.0%)

Mann–Whitney U test was used to test between two groups, * *p* < 0.05 versus 2013, ^††^ *p* < 0.01 and ^†††^ *p* < 0.001 versus 2018.

**Table 2:** Clinical details of preschoolers.

Fiscal Year	2013	2018	2023
Male	43 (49.4%)	62 (52.5%)	36 (53.7%)
Female	44 (50.6%)	56 (47.5%)	31 (46.3%)
Total	87 (100.0%)	118 (100.0%)	67 (100.0%)
d	8.75 ± 4.82	8.00 ± 4.80	9.07 ± 4.87
f	0.32 ± 1.43	0.55 ± 2.04	0.82 ± 2.35
dft	9.07 ± 4.77	8.55 ± 4.78	9.89 ± 4.95

d, decayed; f, filled; dft, decayed and filled teeth.

**Table 3:** Clinical details of elementary school students.

Fiscal Year	2013	2018	2023
Male	26 (72.2%)	24 (63.2%)	24 (45.3%)
Female	10 (27.8%)	14 (36.8%)	29 (54.7%)
Total	36 (100.0%)	38 (100.0%)	53 (100.0%)
d	4.03 ± 3.30	4.26 ± 4.20	4.17 ± 3.91
f	0.19 ± 0.89	0.89 ± 1.52 **	1.08 ± 1.90 **
df	4.22 ± 3.26	5.16 ± 4.25	5.23 ± 4.44
D	1.08 ± 1.71	1.32 ± 1.83	1.96 ± 2.99
M	0.00 ± 0.00	0.00 ± 0.00	0.09 ± 0.69
F	0.06 ± 0.33	0.58 ± 1.22 *	0.30 ± 0.77 *
DMF	1.14 ± 1.78	1.89 ± 2.06 *	2.36 ± 3.13 *

d, decayed; f, filled; df, decayed and filled (primary teeth); D, decayed; M, missing; F, filled; DMF, decayed, missing, and filled (permanent teeth). Mann–Whitney U test was used to test between two groups, * *p* < 0.05 and ** *p* < 0.01 versus 2013.

**Table 4:** Clinical details of junior and senior high school students.

Fiscal Year	2013	2018	2023
Male	2 (33.3%)	5 (71.4%)	3 (42.9%)
Female	4 (66.7%)	2 (28.6%)	4 (57.1%)
Total	6 (100.0%)	7 (100.0%)	7 (100.0%)
D	1.50 ± 1.29 (n = 4)	6.14 ± 6.84	3.71 ± 3.77
M	0.00 ± 0.00 (n = 4)	0.00 ± 0.00	0.00 ± 0.00
F	0.00 ± 0.00 (n = 4)	1.14 ± 2.19	1.43 ± 2.15
DMF	1.50 ± 1.29 (n = 4)	7.29 ± 6.58	5.14 ± 3.67

D, decayed; M, missing; F, filled; DMF, decayed, missing, and filled (permanent teeth).

## Data Availability

The original contributions presented in the study are included in the article, further inquiries can be directed to the corresponding author.
